# *Campylobacter* antimicrobial resistance in Peru: a ten-year observational study

**DOI:** 10.1186/1471-2334-12-193

**Published:** 2012-08-16

**Authors:** Simon Pollett, Claudio Rocha, Rito Zerpa, Lilian Patiño, Augusto Valencia, Máximo Camiña, José Guevara, Martha Lopez, Nancy Chuquiray, Eduardo Salazar-Lindo, Carlos Calampa, Martín Casapia, Rina Meza, Maruja Bernal, Drake Tilley, Michael Gregory, Ryan Maves, Eric Hall, Franca Jones, C Sofia Arriola, Marieke Rosenbaum, Juan Perez, Matthew Kasper

**Affiliations:** 1 Bacteriology Department, U.S Naval Medical Research Unit-6 (NAMRU-6), Venezuela Ave, Block 36, Callao 2, Lima, Peru; 2 Instituto Nacional de Salud del Niño, Lima, Peru; 3 Hospital Nacional Docente Madre-Niño, Lima, Peru; 4 Hospital de Emergencias Pediátricas, Lima, Peru; 5 Hospital Nacional Daniel A.Carrion, Lima, Peru; 6 Laboratorio Servisalud, Cusco, Peru; 7 EsSalud Hospital Alberto Sabogal Sologuren, Lima, Peru; 8 Laboratorio Gastrolab, Lima, Peru; 9 Hospital Apoyo de Iquitos, Iquitos, Peru; 10 Hospital Regional, Iquitos, Peru; 11 Facultad de Medicina Veterinaria, Universidad de San Marcos, San Marcos, Peru

**Keywords:** *Campylobacter*, Antibiotic resistance, Fluoroquinolones, Macrolides, Peru

## Abstract

**Background:**

*Campylobacter jejuni* and *Campylobacter coli* are food-borne pathogens of great importance and feature prominently in the etiology of developing world enteritis and travellers’ diarrhoea. Increasing antimicrobial resistant *Campylobacter* prevalence has been described globally, yet data from Peru is limited. Our objective was to describe the prevalence trends of fluoroquinolone and macrolide-resistant *C. jejuni* and *C. coli* stool isolates from three regions in Peru over a ten-year period.

**Methods:**

Surveillance for enteric pathogens was conducted in Lima, Iquitos and Cusco between 2001 and 2010. *Campylobacter* stool isolates were tested for susceptibilities to ciprofloxacin, azithromycin and erythromycin. Susceptibilities were reviewed for 4652 isolates from Lima ( *n* = 3419), Iquitos ( *n* = 625) and Cusco ( *n* = 608).

**Results:**

Comparing the study periods of 2001-2005 and 2006-2010, prevalence of ciprofloxacin-resistant *C. jejuni* isolates rose in the study areas of Lima (73.1% to 89.8%, *p* < 0.001) and Iquitos (24.1% to 48.9%, *p* < 0.001). Ciprofloxacin-resistant *C. coli* rates also increased in Lima (48.1% to 87.4%, *p* < 0.001) and Cusco (10.0% to 65.9%, *p* = 0.005). Small but significant increases in azithromycin-resistant and erythromycin-resistant *C. jejuni* prevalence were noted in Iquitos (2.2% to 14.9%, *p* < 0.001; 3.2% to 14.9%, *p* = 0.002), and erythromycin-resistant *C. coli* rates increased in Lima (0.0% to 5.3%, *p* = 0.038). The prevalence of *C. jejuni* isolates resistant to both ciprofloxacin and azithromycin increased in Iquitos (0.3% to 14.9%, p < 0.001) and Lima (0.3% to 1.6%, p = 0.011), and prevalence of *C. jejuni* isolates resistant to both ciprofloxacin and erythromycin rose in Iquitos (0.0% to 14.9%, p < 0.001). Ciprofloxacin and erythromycin resistant *C. coli* prevalence increased in Lima (0.0% to 5.3%, p = 0.034).

**Conclusions:**

These results have implications for the empirical management of enterocolitis in Peru. Ongoing surveillance is essential to guide appropriate antimicrobial use in this setting. Local epidemiological studies to explore the relationship between increasing antimicrobial resistance and agricultural or human antibiotic use may be valuable.

## Background

*Campylobacter jejuni* and *Campylobacter coli* are food-borne zoonotic pathogens that play a major role in the etiology of human bacterial enterocolitis globally [1]. *Campylobacter* is a notable pathogen in developing-world childhood gastroenteritis illness [2], immunocompromised patients [3] and traveller’s diarrhea [4]. The spectrum of campylobacteriosis can range from mild to severe, particularly in the immunosuppressed, and may be complicated by neurological and rheumatological sequelae [1].

Fluoroquinolones, such as ciprofloxacin, and macrolides, namely azithromycin and erythromycin, are the antibiotic agents of choice for campylobacteriosis [1]. *Campylobacter* resistance to these agents has long been described, particularly resistance to fluoroquinolones [5,6]. The genetic determinants of fluoroquinolone resistance are well characterized, with only a single-step mutation in the *gyrA* gene required to confer phenotypic resistance [7]. The last two decades have seen a striking rise in quinolone resistance in settings as diverse as North America, Europe, the Middle East, Africa and Asia [6,8-11]. While quinolone resistance in Latin America is documented, data from Peru is limited [12,13].

The therapeutic implications of quinolone resistance in campylobacteriosis are significant, as they are widely used as empirical first-line antimicrobials for childhood, travel and other febrile gastrointestinal syndromes in both developed and developing countries, including Peru [14,15]. Such resistance trends are also concerning as they may implicate widespread human and animal quinolone use in driving escalating resistance [16,17]. Given such potential clinical and public health issues, comprehensive resistance surveillance for this organism is a priority in this region. The objective of this study was to describe the trends in fluoroquinolone and macrolide resistance over a ten-year period in multiple regions of Peru.

## Methods

### Study sites

Surveillance for enteric pathogens in Peru was conducted at nine hospital and community medical laboratories throughout Lima (Instituto Nacional de Salud del Niño, Hospital Nacional Docente Madre-Niño, Hospital de Emergencias Pediátricas, EsSalud Hospital Alberto Sabogal Sologuren, Laboratorio Gastrolab and Hospital Nacional Daniel A.Carrion), Cusco (Laboratorio Servisalud) and Iquitos (Hospital Apoyo de Iquitos and Hospital Regional). These laboratories serve three diverse regions in Peru. Lima is a coastal city with an estimated population of 8.5 million persons. Cuzco is a regional center in the Andes with a population of around 1.2 million and Iquitos is an isolated urban hub amidst the Amazon, with some 370,000 residents [18]. Laboratories in these latter two provincial cities likely serve a significant surrounding catchment population. All laboratories are public, except that in the Cusco study site. Surveillance occurred from January 2001 to December 2010.

### Bacterial identification

Isolates identified as *Campylobacter* spp *.* from stool at study sites as part of routine clinical practice were placed into Cary-Blair transport media and stored at 4°C at the study site. Every fifteen days, isolates from each study site were transported to the U.S Naval Medical Research Unit-6 (NAMRU-6) laboratory at 4°C for confirmation of identification, speciation and antimicrobial susceptibility testing. Bacterial isolates received from study sites were re-cultured and re-isolated according to methods described elsewhere [19,20]. Briefly, *Campylobacter* blood-free selective agar media with ECR 0155 supplement (Oxoid, Cambridge, UK) were inoculated and incubated in a microaerophilic environment with a gas mixture of 10% CO2, 5% O2, and 85% N2 in anaerobic jars at 42°C for 24 to 48 hours. *Campylobacter jejuni* was differentiated from other *Campylobacter* species, including *C. coli*, by hippurate hydrolysis assay.

### Antimicrobial susceptibility testing

Antimicrobial susceptibility disk diffusion testing with ciprofloxacin, azithromycin and erythromycin was performed according to CLSI guidelines, with disk diffusion zones for all isolates interpreted as per the most recent edition of these guidelines [20]. A stock *Staphylococcus aureus* strain ATCC®25923 (ATCC, Virginia, USA) cultured on Mueller Hinton Agar (Oxoid, Cambridge, UK) at 35°C to 37°C was used as a control for susceptibilities testing conditions. Resistance prevalence was compared between the two time periods of 2001 to 2005 and 2006 to 2010 using a Chi-square or Fisher’s exact test (Statistical Package for the Social Sciences, Version 17.0, New York, USA).

### Ethical considerations

The testing of these bacterial isolates was determined not to be human subjects research by the Institutional Review Board of NAMRU-6.

## Results

Susceptibilities were reviewed for a total of 4652 non-duplicate isolates from Lima (n = 3419), Iquitos (n = 625) and Cuzco (n = 608). Of all isolates, 82.9% (3856/4652) were identified as *C. jejuni*, followed by *C. coli* (11.9%, 554/4652) and other related or non-identified species (7%, 242/4652), including *C. lari* and *C. laridis*. Prevalence trends of antimicrobial resistance to ciprofloxacin, azithromycin and erythromycin by time and region in *C. jejuni* and *C. coli* isolates are summarised in Table 1 and Table 2 respectively.

**Table 1:** **Prevalence of antimicrobial-resistant* Campylobacter jejuni *over time and by region**

		**N° Total**	***Campylobacter jejuni***								
			**AZM**			**CIP**			**E**		
			**N° Resis**	**%**	**p-value**	**N° Resis**	**%**	**p-value**	**N° Resis**	**%**	**p-value**
Cusco	2001-2005	62	0	0.0	1.000	45	72.6	0.051	0	0.0	1.000
	2006-2010	465	2	0.4		385	82.8		2	0.4	
Iquitos	2001-2005	316	7	2.2	<0.001	76	24.1	<0.001	10	3.2	0.002
	2006-2010	47	7	14.9		23	48.9		7	14.9	
Lima	2001-2005	644	9	1.4	0.399	471	73.1	<0.001	8	1.2	0.264
	2006-2010	2322	45	1.9		2084	89.8		45	1.9	

AZM = azithromycin, CIP = ciprofloxacin, E = erythromycin.

**Table 2:** **Prevalence of antimicrobial-resistant* Campylobacter coli *over time and by region**

		**N° Total**	***Campylobacter coli***								
			**AZM**			**CIP**			**E**		
			**N° Resis**	**%**	**p-value**	**N° Resis**	**%**	**p-value**	**N° Resis**	**%**	**p-value**
Cusco	2001-2005	10	0	0.0	-	1	10.0	0.005	1	10.0	0.196
	2006-2010	41	0	0.0		27	65.9		0	0.0	
Iquitos	2001-2005	205	2	1.0	1.000	40	19.5	0.686	6	2.9	0.287
	2006-2010	10	0	0.0		3	30.0		1	10.0	
Lima	2001-2005	81	4	4.9	1.000	39	48.1	<0.001	0	0.0	0.038
	2006-2010	207	12	5.8		181	87.4		11	5.3	

AZM = azithromycin, CIP = ciprofloxacin, E = erythromycin.

Between 2001-2005 and 2006-2010, significant changes in fluoroquinolone-resistant *C. jejuni* prevalence were noted in the Lima and Iquitos regions (Table 1). Significant changes in ciprofloxacin-resistance rates between 2001-2005 and 2006-2010 were observed in *C. coli* isolates from both Lima and Cusco (Table 2). Most strikingly, the prevalence of ciprofloxacin-resistant *C. jejuni* isolates from Lima rose from 42.1% in 2001 (24/57) to 91.1% in 2010 (319/348, p < 0.001).

There was no significant change in macrolide resistance in *C. jejuni* either in the Lima or the Cusco regions between the periods of 2001-2005 and 2006-2010 (Table 1). However, statistically significant increases in *Campylobacter jejuni* resistance to the macrolides azithromycin and erythromycin were seen in the Iquitos region (Table 1). In Lima, a statistically significant rise in erythromycin-resistant *C. coli* isolates was seen over the same time period (Table 2).

The prevalence of *C. jejuni* and *C. coli* isolates resistant to both quinolones and macrolides was also compared between the two study periods, and are presented in Table 3 and Table 4 respectively. Statistically significant increases in the prevalence of multidrug-resistant *C.jejuni* were noted in Iquitos and Lima, and the prevalence of multidrug-resistant *C. coli* increased in Lima.

**Table 3:** **Prevalence of multidrug-resistant*Campylobacter jejuni*over time and by region**

		**N° Total**	***Campylobacter jejuni***					
			**CIP-AZM**			**CIP-E**		
			**N° Resis**	**%**	**p-value**	**N° Resis**	**%**	**p-value**
Cusco	2001-2005	62	0	0	0.605	0	0	0.715
	2006-2010	465	2	0.4		1	0.2	
Iquitos	2001-2005	316	1	0.3	<0.001	0	0	<0.001
	2006-2010	47	7	14.9		7	14.9	
Lima	2001-2005	644	2	0.3	0.011	5	0.8	0.106
	2006-2010	2322	37	1.6		38	1.6	

AZM = azithromycin, CIP = ciprofloxacin, E = erythromycin.

**Table 4:** **Prevalence of multidrug-resistant*Campylobacter coli*over time and by region**

		**N° Total**	***Campylobacter coli***					
			**CIP-AZM**			**CIP-E**		
			**N° Resis**	**%**	**p-value**	**N° Resis**	**%**	**p-value**
Cusco	2001-2005	10	0	0	-	0	0	-
	2006-2010	41	0	0		0	0	
Iquitos	2001-2005	205	2	1	0.754	2	1	0.754
	2006-2010	10	0			0	0	0	
Lima	2001-2005	81	2	2.5	0.238	0	0	0.034	
	2006-2010	207	12	5.8		11	5.3		

AZM = azithromycin, CIP = ciprofloxacin, E = erythromycin.

## Discussion

The significant increase in fluoroquinolone-resistant *Campylobacter* prevalence described here reflects similar trends noted in both the developed and developing world over the last two decades [5,6]. These findings are supported by recent but limited data from Peru which show a comparatively high prevalence (63-78%) [12]. Resistance rates in other South American countries vary from 14% to 72% [12,13,21,22]. Resistance elsewhere in the world is also variable, with rates as high as 29% in Africa, 41% in North America, 80% in Europe, and 93-100% in Asia [6,8-10,23].

Fluoroquinolone resistance in *Campylobacter* species is primarily mediated by mutations in *gyrA,* a gene which expresses DNA gyrase, the target of quinolones [7]. Phenotypic resistance requires a one-step mutation only [7], and therapeutic use of fluoroquinolone antibiotics has been shown to be associated with subsequent isolation of quinolone-resistant *Campylobacter* in humans [16,17,24,25]. As such, the widespread human use of fluoroquinolones may at least partly contribute to emerging quinolone resistance [16,17,24].

Human quinolone use may be a factor in the trends described here. Fluoroquinolones are frequently used in Peru, with ciprofloxacin identified as the most common antibiotic prescribed in a study evaluating prescribing practices in an urban outpatient medical centre in Lima [26]. Whether the very high quinolone resistance prevalence noted in Lima reflects a comparatively greater clinical use of ciprofloxacin in this region compared to others is unclear. Like many other developing countries, regulation of these and other antibiotics in Peru is limited [27] and self-medication with antimicrobials in the Peru community is well recognised [28]. Antibiotics may be purchased without prescription, with as much as 29% obtained without medical authorisation [28]. Furthermore, the quality of antibiotic prescription practices in Peru is variable [26,28].

Fluoroquinolone-resistant *Campylobacter* have been isolated in patients with no prior history of quinolone use [29]. Moreover, humans are not generally a reservoir of *Campylobacter* infection for other humans. It has thus been suggested that there are other causes for rising resistance [17]. The intensive use of quinolones in the food animal industry, especially poultry, and transmission of resistant organisms via the food chain is thought to be another cause of rising quinolone resistance [3,5,16,24,30], although the relationship is complex and causality is difficult to determine [24,31-35]. As such, agricultural (rather than human) quinolone use may play a greater role in the development of resistant *Campylobacter* in humans, particularly as resistance prevalence increased in many countries only after the introduction of quinolones into animal use [24]. The World Health Organisation has called for the restriction of the use of antimicrobials in agriculture due to concerns of their role in *Campylobacter* and other human enteropathogen resistance [3]. Countries such as the United States have banned the use of quinolones in poultry for this reason [10].

It is possible the food chain may contribute to the high prevalence of resistance described here. Quinolone-resistant *Campylobacter* have been isolated from poultry in Peruvian shanty towns [36], live birds sold in Peruvian markets [37], and from food animals in neighbouring Latin American countries [13,27,37,38]. Ciprofloxacin resistant *E. coli* has also been isolated from both food animals and commercial meat samples in Peru [27] (C.S.Arriola, unpublished data).

Animal antibiotic use is not currently restricted or prohibited in Peru (C.S.Arriola, personal communication). It is mandated to register all antibiotics imported into Peru with the Peruvian National Tax Administration (known by its Spanish-language acronym ‘SUNAT’), including those used in agriculture. Typical agricultural fluoroquinolones such as enrofloxacin are among such imported antibiotics [39]. While antibiotics must be licensed for animal use in Peru with the Peruvian Agency of Animal Health (known by its Spanish-language acronym ‘SENASA’) [40], the nature and extent of their use in food animals is not directly monitored or measured (C.S.Arriola personal communication). Enrofloxacin and norfloxacin are known to be used as therapeutic and prophylactic antibiotics, respectively, in some food animals in Peru (C.S.Arriola, personal communication), although the extent of this practice is unclear. Further studies, including detailed surveillance of animal antibiotic use and measurement of the prevalence of quinolone-resistant *Campylobacter* in commercially raised food animals, would be valuable to assess the role of the food chain in the development of quinolone-resistant *Campylobacter* in this region. Such studies would be particularly interesting to undertake in the Lima region, where the prevalence of quinolone resistance increased the most during our study period (42.1% in 2001 to 91.1% in 2010, p < 0.001) and where food animals may be more intensively farmed due to a greater population density.

International travel has also been identified as a risk factor for clinical isolation of quinolone-resistant *Campylobacter*[17,29]. It is unclear what role, if any, this risk factor plays in this population.

Macrolide resistance in this study was low overall, although a small but significant increase in prevalence of macrolide-resistance *Campylobacter* was noted in Iquitos (in the case of *C. jejuni*) and Lima (in the case of *C. coli*). Limited comparative data from Peru quotes a macrolide-resistant *Campylobacter* prevalence of 17% [12]. Literature from other Latin American countries reports a highly variable prevalence from 0% to 58.6% [12,13,22,41,42]. Caution is needed when interpreting these comparative prevalence rates, as many of them incorporate animal data. The prevalence of macrolide-resistant *Campylobacter* is variable but generally uncommon in many other parts of the world by comparison to fluoroquinolones [5,6,21,43,44]. However, higher rates of resistance have been noted in some regions including Africa [45], Europe [45] and Australasia [6,24,45]. Where significant macrolide resistance exists, it is usually most prevalent in non*-jejuni* isolates [6,43,45].

The overall low prevalence of macrolide resistance seen in Peru and other parts of the world is thought to reflect a lower mutation rate and a longer requisite macrolide exposure time needed to develop resistance. [5]. Macrolides are used less commonly than fluoroquinolones in Peru[26], and this also may account for the low rates of resistance here, although understanding of the link between human macrolide use and macrolide resistance rates is still evolving [24,46]. It is unknown whether the small increase in prevalence in Lima and Iquitos over the study period reflects a possible recent increase in human macrolide use in those study areas.

Like fluoroquinolones, macrolides are used extensively in food animals in many countries [6,24], including Peru (C.S.Arriola, unpublished data). The food chain may play a role in the etiology of human macrolide-resistant *Campylobacter* spp, although the strength of the association is unclear [24,45]. Macrolide-resistant *Campylobacter* have been isolated from food animals in countries within South America and beyond, particularly in swine [5,6,42]. Whether the rise in prevalence in Lima and Iquitos reflects animal macrolide use is a question that requires further study.

Of all the study regions, only Iquitos showed a statistically significant increase in resistance to all three antibiotics studied. The reason for this is unclear. Possible causes could include an increase in the human or animal use of all three of these antibiotics in this region over the study period. Surveillance of medical and agricultural antibiotic usage in this region, and possibly human-animal strain linkage studies would be needed to approach this question. We are unaware of any other factors in particular that may influence enteric organism antibiotic resistance in the Iquitos region specifically.

These results have significant therapeutic implications for the empirical management of enterocolitis in Peru, including those with a diarrheal syndrome during or after travel to Peru. Fluoroquinolones are commonly used first-line as the empirical management of both developing world childhood dysentery [15] and travellers’ diarrhea [14]. Given the important role *Campylobacter* plays in the etiology of these clinical syndromes, the risk of antibiotic failure with empirical fluoroquinolone use in these settings may be significant based on the resistance prevalence we have described here.

While the absolute number of multidrug-resistant *Campylobacter* isolates was low, the increasing prevalence of *Campylobacter* spp resistant to both fluoroquinolones and macrolides in the Lima and Iquitos areas is concerning, as these are the two classes of antibiotics usually used to treat campylobacteriosis, with few to no alternative oral therapeutic options available should these agents fail [1], particularly in a developing world setting.

There are several potential limitations in this study. Interpretation of resistance trends is limited by the low surveillance activity and isolate referral rates in some regions, with a resultant loss of statistical power. However, analysing the trends by five-year periods allowed us to observe some statistically significant trends. The number of referring laboratories increased over time, and data on patient characteristics such as age, sex, socioeconomic status, occupation, travel history and prior antibiotic use was not collected. Changes in the study population with respect to such variables could affect resistance prevalence over time. Measuring these characteristics would be valuable in future studies to assess the generalizability of antimicrobial resistance trends described here, to explore associations of these characteristics with *Campylobacter* resistance, and to identify sociodemographic or biological predictors of antimicrobial-resistant *Campylobacter*. Measurement of laboratory catchment population size, changes in this catchment population size, and faecal sample submission rates would also be valuable in future studies for the interpretation of resistance trends.

New, sensitive and specific molecular techniques for the identification of *Campylobacter* species have emerged and these may be useful for future surveillance studies [47]. In addition, significant interlaboratory variation in macrolide susceptibilities have been described, particularly with non-microdilution methods [20,48]. While we followed CLSI guidelines for the disk-diffusion method in testing macrolide and fluoroquinolone susceptibilities, discrepancy between this technique and reference standard dilutional assays have been described in a recent study. Confirming all susceptibilities by dilutional assay may be preferable for future antimicrobial resistance surveillance [20,49].

## Conclusions

In conclusion, significant changes in the prevalence of antibiotic-resistant *Campylobacter* have been observed in Peru, most notably with resistance to fluoroquinolones. Multi-drug resistant *Campylobacter* appears to be emerging in some areas of Peru. This has implications for the empirical management of diarrheal infections in this region. Ongoing surveillance of antimicrobial resistance in all regions is essential to guide appropriate antimicrobial use. There is a potential link between emerging antimicrobial resistance and agricultural antibiotic use, and local epidemiological studies to explore this relationship would be valuable. More stringent monitoring and regulation of human and animal antimicrobial use, particularly quinolone use, is warranted in this region.

## Abbreviations

AZM: Azithromycin; CIP: Ciprofloxacin; E: Erythromycin; CLSI: Clinical and Laboratory Standards Institute.

## Competing interests

All authors declare no conflict of interest.

## Authors’ contributions

SP, CR, JP and MK analyzed the data. SP, MK, MG, DT, MR and SA helped draft the manuscript. RM, EH and FJ contributed to study design. RM, MB, MG and MK coordinated/performed all microbiological assays at NAMRU-6. RZ, LP, AV, MC, JG, ML, NC, EL, CC and MC coordinated study procedures and microbiological assays at peripheral study sites. All authors read and approved the final manuscript.

## Disclaimer

The views expressed in this article are those of the authors and do not necessarily reflect the official policy or position of the Department of the Navy, Department of Defense, nor the U.S. Government. Several of the authors are military service members. This work was prepared as part of our official duties. Title 17 U.S.C. §105 provides that ‘Copyright protection under this title is not available for any work of the United States Government.’ Title 17 U.S.C. §101 defines a U.S. Government work as a work prepared by a military service member or employee of the U.S. Government as part of that person’s official duties.

## Pre-publication history

The pre-publication history for this paper can be accessed here:

http://www.biomedcentral.com/1471-2334/12/193/prepub
